# Carotenoids in Cancer Apoptosis—The Road *from Bench to Bedside* and Back

**DOI:** 10.3390/cancers12092425

**Published:** 2020-08-26

**Authors:** Lenka Koklesova, Alena Liskova, Marek Samec, Constanze Buhrmann, Samson Mathews Samuel, Elizabeth Varghese, Milad Ashrafizadeh, Masoud Najafi, Mehdi Shakibaei, Dietrich Büsselberg, Frank A. Giordano, Olga Golubnitschaja, Peter Kubatka

**Affiliations:** 1 Department of Obstetrics and Gynecology, Jessenius Faculty of Medicine, Comenius University in Bratislava, 036 01 Martin, Slovakia; koklesova.lenka@gmail.com (L.K.); liskova80@uniba.sk (A.L.); marek.samec@gmail.com (M.S.); 2 Musculoskeletal Research Group and Tumour Biology, Chair of Vegetative Anatomy, Institute of Anatomy, Faculty of Medicine, Ludwig-Maximilian-University Munich, D-80336 Munich, Germany; constanze.buhrmann@med.uni-muenchen.de (C.B.); mehdi.shakibaei@med.uni-muenchen.de (M.S.); 3 Department of Physiology and Biophysics, Weill Cornell Medicine in Qatar, Education City, Qatar Foundation, Doha 24144, Qatar; sms2016@qatar-med.cornell.edu (S.M.S.); elv2007@qatar-med.cornell.edu (E.V.); dib2015@qatar-med.cornell.edu (D.B.); 4 Department of Basic Science, Faculty of Veterinary Medicine, University of Tabriz, 51368 Tabriz, Iran; dvm.milad1994@gmail.com; 5 Radiology and Nuclear Medicine Department, School of Paramedical Sciences, Kermanshah University of Medical Sciences, 67146 Kermanshah, Iran; najafi_ma@yahoo.com; 6 Department of Radiation Oncology, University Hospital Bonn, Rheinische Friedrich-Wilhelms-Universität Bonn, 53127 Bonn, Germany; frank.giordano@ukbonn.de; 7 Predictive, Preventive Personalised (3P) Medicine, Department of Radiation Oncology, University Hospital Bonn, Rheinische Friedrich-Wilhelms-Universität Bonn, 53127 Bonn, Germany; 8 Department of Medical Biology, Jessenius Faculty of Medicine, Comenius University in Bratislava, 03601 Martin, Slovakia

**Keywords:** carotenoids, phytochemicals, plant substances, apoptosis, programmed cell death, intrinsic pathway, extrinsic pathway, anti-cancer therapy, chemoprevention, nutritional supplement, vitamins, cancer cells, ROS, predictive preventive personalized medicine (PPPM/3PM), patient stratification, individualized patient profiling, complementary medicine, targeted treatment, nanotechnology, bioavailability, solubility, sensitizers

## Abstract

An incidence and mortality of cancer are rapidly growing worldwide, especially due to heterogeneous character of the disease that is associated with irreversible impairment of cellular homeostasis and function. Targeting apoptosis, one of cancer hallmarks, represents a potent cancer treatment strategy. Carotenoids are phytochemicals represented by carotenes, xanthophylls, and derived compounds such as apocarotenoids that demonstrate a broad spectrum of anti-cancer effects involving pro-apoptotic signaling through extrinsic and intrinsic pathways. As demonstrated in preclinical oncology research, the apoptotic modulation is performed at post-genomic levels. Further, carotenoids demonstrate additive/synergistic action in combination with conventional oncostatic agents. In addition, a sensitization of tumor cells to anti-cancer conventional treatment can be achieved by carotenoids. The disadvantage of anti-cancer application of carotenoids is associated with their low solubility and, therefore, poor bioavailability. However, this deficiency can be improved by using nanotechnological approaches, solid dispersions, microemulsions or biofortification that significantly increase the anti-cancer and pro-apoptotic efficacy of carotenoids. Only limited number of studies dealing with apoptotic potential of carotenoids has been published in clinical sphere. Pro-apoptotic effects of carotenoids should be beneficial for individuals at high risk of cancer development. The article considers the utility of carotenoids in the framework of 3P medicine.

## 1. Introduction

Cancer is characterized by uncontrolled growth of abnormal cells in the organism. An initial stage of cancer is irreversible and leads to increased tumor cell proliferation [1]. According to Global Cancer Statistics (GLOBOCAN) 2018, cancer incidence and mortality are rapidly growing worldwide [2]. Carcinogenic factors can be classified into three groups: primary determining factors (chemical substances, physical agents, viruses), secondary determining factors (hereditary determinism), and favoring factors (geographic factors, nutrition, sex, age, etc.). These carcinogenic factors can contribute to cancer development characterized by disturbed cellular homeostasis by hyperplastic, dysplastic or regenerative alterations [3]. Moreover, carcinogenesis is commonly associated with DNA damage as a result of exposure to various exogenous (ultraviolet rays, radiation, pollution, smoking, and stress) and endogenous agents (lack of apoptotic function, genetic mutation, oxidative stress, and hypoxia) [4,5].

Under physiological conditions, apoptosis is one type of programmed cell death, where strictly controlled pathways are responsible for a destruction of unwanted, old, and damaged cells [6]. In the case of cancer, tumor cells avoid apoptosis in order to survive. Deregulation of apoptosis-inducing pathways can consequently lead to the expansion of tumor cells with the potential for metastatic spread [7,8]. Due to the frequent acquisition of cancer resistance to conventional drugs, the discovery of new anticancer agents with pro-apoptotic activities that have potential for use in combined anti-cancer therapy is undoubtedly important [9,10,11,12].

Carotenoids (a group that comprises more than 700 different documented molecules including carotenes, xanthophylls, and their derived compounds called apocarotenoids [13]), are present in different sources such as plants, fungi, algae, birds, fish flesh, bacteria, microalgae, and yeasts [14,15]. Carotenoids represent a group of phytochemicals that exert beneficial effects on human health. Dietary carotenoids have been described to prevent diseases by enhancement of the immunity system [16,17]. Some carotenoids demonstrate a role as precursors of vitamin A that is crucial for vision, growth, cell differentiation, and other physiological processes [18]. Moreover, the antioxidant capacity of carotenoids protects humans from weakened immune response, premature aging, cardiovascular diseases, and arthritis [19]. Some carotenoids have also revealed anti-inflammatory (astaxanthin) [20], radiation protective (lycopene) [21] or anti-obesity (fucoxanthin) effects [22]. It is not surprising that carotenoids have also gained attention in cancer research [23,24]. Various anti-cancer effects, including cell cycle arrest closely associated with apoptosis [25], anti-metastatic, anti-invasive [26], anti-angiogenic [27], and anti-proliferative [28] effects, the inhibition of tumor growth [29] and the reduction of risk of cancer [30], have been observed using carotenoid treatments. Further, extensive preclinical cancer research has revealed the pro-apoptotic potential of carotenoids through targeting various signaling pathways involved in extrinsic and intrinsic pathways [31,32], metabolic apoptotic processes [33], and epigenetic modulation [34].

Evidence from clinical anticancer research also focuses on fenretinide or bexarotene, synthetic retinoids that belong to the apocarotenoids group. Despite the fact that fenretinide exerted anti-cancer effects in preclinical cancer models [35,36], the results of more recent clinical studies are not clear [37,38,39]. On the contrary, combinations of bexarotene with chemotherapeutic drugs were found to be safe and well tolerated [40,41] and all-*trans*-retinoic acids tretionin revealed an effective and safe impact on field cancerization [42]. However, there is lack of evidence of pro-apoptotic effects of carotenoids in clinical practice. Above all, preclinical evidence of carotenoid associated with apoptotic molecules and signaling pathways represents the basis for further research and potential future use of carotenoids targeting apoptotic processes to improve cancer management in terms of specific targeting and personalized cancer therapy.

### 1.1. Aim of the Study

This review focuses on current evidence from both preclinical and clinical research of carotenoids’ activities in the induction of programmed cell death. A special part of the review summarizes antitumor effects of carotenoids based on the induction of apoptosis, targeting signal molecules involved in intrinsic or extrinsic apoptotic pathways.

### 1.2. Source of the Data

Data were obtained from the English-language biomedical literature by the use of “apoptosis” or “intrinsic pathway” or “extrinsic pathway” or “cell-death” and “carotenoids“ or “carotenes” or “xanthophylls“ or “apocarotenoids” or “retinoids” or other associated terms as either a keyword or medical subject heading (MeSH) term in searches of the PubMed bibliographic database. In the special part concerning the antitumor effect of carotenoids, we emphasize using of the most recent scientific papers from the years January 2016–August 2020.

## 2. Insights into the Cell Death Mechanisms

Cell death is an essential event associated with homeostasis in multicellular organisms [43]. Elimination of unwanted cells is widely detected during metamorphosis, embryogenesis as well as during pathogenetic conditions. This fundamental process is classified according to morphological, enzymological, functional or immunological characteristics [44]. Generally, apoptosis and necrosis are the most widely used classification categories of cell death defined by morphological criteria. Necrosis is an uncontrolled mode of cell death as a consequence of severe and devastating trauma [45]. Like necrosis, necroptosis is an alternative form of cell death that displays the specifics of necrosis. However, necroptosis is initiated by similar pathways that are involved in the apoptosis (e.g., ligation of tumor necrosis factor receptor 1 (TNFR1) receptor) and thus, it represents a category of necrosis that is highly regulated [45,46]. Importantly, necroptosis can be actively suppressed by components of the death receptor pathways of apoptosis such as caspase-8, Fas-associated death domain protein (FADD), and FADD-like IL-1β-converting enzyme inhibitory protein (FLIP) [47]. Programmed cell death (PCD) represents an important concept, which is defined as a genetically regulated cascade leading to controlled cell removal. PCD involves two major types of cell death: apoptosis and autophagy. Autophagy as a distinct mode of cell death, is associated with the generation of energy and metabolites by digestion of intracellular macromolecules and organelles. In long term deprivation of nutrients, all substrates are digested that result in autophagy associated cell death [45].

Undoubtedly, apoptosis is the best-characterized form of PCD [48]. Apoptosis exerts specific morphological characteristics such as cytoplasmatic cell shrinkage, chromatin condensation and fragmentation of DNA, budding of the plasma membrane and exposure of phosphatidylserine on the outer plasma membrane. Apoptotic cascade depends on the family of proteases known as caspases that cleave intracellular substrates resulting in the morphological and biochemical fluctuations, typical for apoptosis. Caspases are produced as inactive enzymes (pro-caspases) and their activation is performed via cleavage of an enzyme pro-domain, usually by other caspase [49]. From the functional view, 2 types of caspases exist: initiator caspases (caspase-2, -8, -9, -10) and executor caspases (caspase-3, -6, -7). Initiator caspases are activated by auto-proteolysis. Once activated, the initiator caspases are able to cleave off the executor caspases resulting in final apoptotic cell death [50].

Under physiological conditions, apoptosis and cellular senescence are two primarily tumor-suppressive mechanisms, the role of which is to ensure the health during early and reproductive stages of life [51]. Moreover, apoptosis is considered as a vital component of various processes including normal cell turnover, proper development and functioning of the immune system, hormone-dependent atrophy, embryonic development and chemical-induced cell death [52]. The prevention of neoplastic transformation is a key function of apoptosis in the cell. Deregulation of apoptotic control leads to the survival of cancer cells and demonstrates a crucial event associated with the accumulation of mutations and promotion of invasiveness, angiogenesis, and other cancer-related processes [8]. Cancer is classified according to several hallmarks and evasion of cell death plays an essential role in tumor development [53,54].

Many aberrations of originally optimal apoptotic flow including alteration of intrinsic and extrinsic pathways lead to the suppression of apoptosis and acquisition of resistance to cell death. Disruption of the balance between anti-apoptotic and pro-apoptotic proteins, impaired death receptors signaling, disturbed p53 function or defects in optimal caspase function are detected in tumor cells [55].

**Figure 1:**
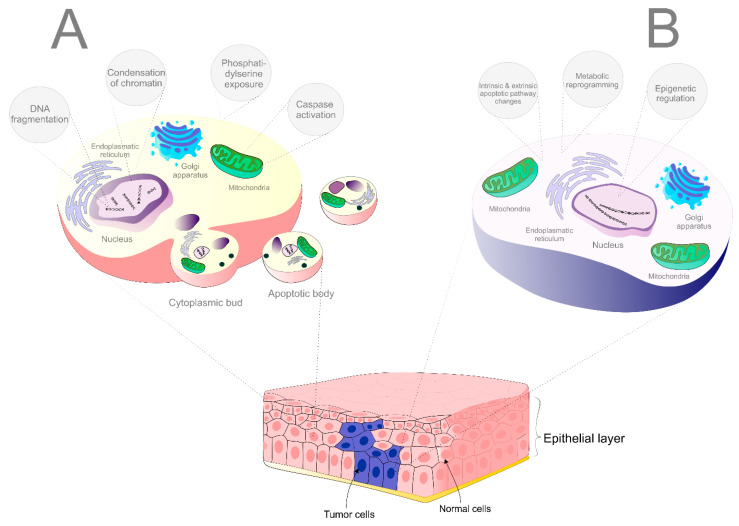
The difference between apoptosis in normal and tumor cells. (**A**) Apoptosis in physiological state (leading to cell fragmentation, degradation, and resorption). (**B**) Evasion of cell death in cancer (leads to the survival of the tumor cells).

The modulation of apoptosis, targeting either intrinsic or extrinsic pathway, represents a promising way to treat cancer because the use of cells’ own mechanism for the eradication of the tumor is a very effective approach in the oncology-related research field [8]. According to the mechanism of action, therapeutic strategies targeting cell death cascade can be classified into two different types: stimulation of the pro-apoptotic molecules, or suppression of anti-apoptotic molecules [56]. Potential targets that have been studied include B-cell lymphoma 2 (Bcl-2)/ B-cell lymphoma-extra large (Bcl-xl) inhibitors, myeloid cell leukemia 1 (MCL-1) inhibitors, inhibitors of apoptosis proteins (IAPs) and second mitochondria-derived activator of caspases (SMAC) mimetics, death receptors (DR) agonists or strategies targeting p53 (mouse double minute 2 homolog (Mdm2) inhibitors or restoring the activity of p53) [11]. Due to the fact that apoptosis causes only minimal inflammatory reaction and tissue damage is also reduced, a therapeutic strategy targeting apoptotic pathways is a promising way for intervention against cancer [57,58]. The differences between apoptosis in physiological condition and avoidance of apoptosis of tumor cells are described in Figure 1.

## 3. A Molecular View and Pathways Involved in Apoptosis

Due to the deregulation of apoptosis caused by the disbalance between pro-apoptotic and anti-apoptotic protein regulators, it seems that targeting the molecules and pathways involved in apoptosis and sensitization of the apoptosis-resistant cancer cells could be a good choice for therapeutic strategies [8,11,59,60]. Apoptosis can be induced by different mechanisms including extrinsic and intrinsic (mitochondrial dependent) pathways, oxidative stress, metabolic and epigenetic regulations associated with various pro-apoptotic and anti-apoptotic molecules, proteins, genes, receptors, etc. [55].

### 3.1. Extrinsic Pathway

The extrinsic pathway, known as DR-dependent pathway, is mediated by an interaction of DRs(TNFR, DR4, DR5, CD95) with their respective ligands (TNF, TNF-related apoptosis inducing ligand (TRAIL), Fas ligand (FasL) (CD95L)) [55,61]. A part of the structure of DR consists of a protein-protein interaction domain called the death domain (DD) [60]. After stimulation of DR by a ligand, DR starts to oligomerize that result in the conformational change leading to its cytoplasmic DD exposure. DD exposed to the cytoplasm is appropriate for the homotypic interaction with other DD-containing proteins as a new binding site of adapter proteins (FADD/ tumor necrosis factor receptor type-1 associated death domain (TRADD)) [61]. Moreover, adapter proteins that bind to pro-caspase-8/-10, create a complex called death-inducing signaling complex (DISC), which cause the auto-activation of adapter proteins [62]. Furthermore, the pro-caspase-8/-10 cleavage causes subsequent activation of downstream effector caspases-3 and -7 that induces the cell death [63].

Not all cells undergo apoptosis only by activating the extrinsic signaling pathway, but additional caspase-8 and -10 activate the protein BH3-interacting-doamin death agonist (BID), which leads to the cleavage and generation of the truncated BH3 interacting domain death agonist (tBID) fragment. tBID in turn activates pro-apoptotic proteins such as Bcl-2-associated X protein (BAX) and Bcl-2 homologous antagonist/killer (BAK) to promote mitochondrial outer membrane permeability (MOMP) and the release of cytochrome c, which leads to the activation of the intrinsic signaling pathway [64]. Therefore, the apoptotic response is a result of a cooperation of extrinsic and intrinsic pathways [64].

### 3.2. Intrinsic Pathway

Several stress conditions such as irradiation, chemotherapeutical agents or different internal stimuli, including irreparable genetic damage, oxidative stress, hypoxia, high concentration of cytosolic Ca^+^, lead to the induction of intracellular signals at mitochondrial level and a response through intrinsic (mitochondria-dependent) apoptotic pathway, the most common mechanism of apoptosis in vertebrates [65,66]. These signals activate BH3-only members of the Bcl-2 family (Bcl-2-interacting mediator of cell death (BIM), BID, Bcl-2 associated agonist of cell death (BAD), Bcl-2-modifying factor (BMF), phorbol-12-myristate-13-acetate-induced protein 1 (Noxa), and p53 upregulated modulator of apoptosis (PUMA)) as well as the proapoptotic proteins BAX and BAK, which neutralizes the Bcl-2, Bcl-xL, and Mcl-1 anti-apoptotic proteins with subsequent disruption of MOMP. Moreover, an intermembrane space of mitochondria includes cytochrome c, SMAC, and high temperature requirement factor A2 (HtrA2/OMI) and the disruption of MOMP cause their spread into the cytosol [67]. An oligomerization of apoptosis protease activating factor-1 (Apaf-1) leads to the formation of a complex called apoptosome that consists of the C-terminal *WD40* region of Apaf-1, cytochrome c, pro-caspase-9 with caspase recruitment domain (CARD) [68]. This complex activates caspase-9 and subsequently its downstream executioner caspases-3, -6, and -7 that cleave the cellular substrates and cause the apoptotic cell death [69].

**Figure 2:**
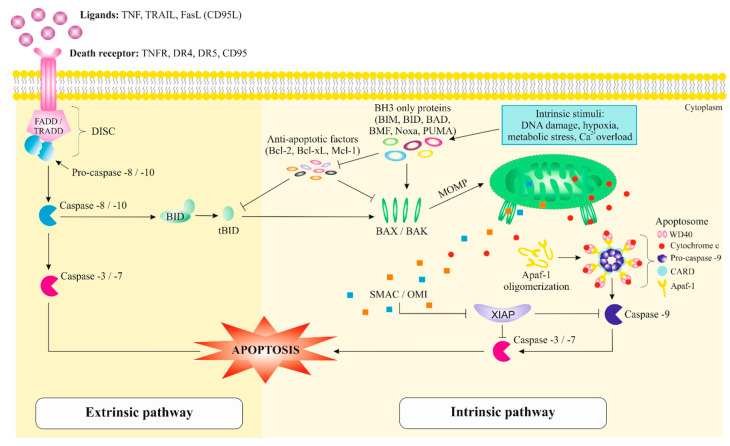
Molecular mechanism of extrinsic and intrinsic pathway involved in apoptosis. Abbreviations: TNF, tumor necrosis factor; TRAIL, TNF-related apoptosis-inducing ligand; FasL, Fas ligand; TNFR, tumor necrosis factor receptor; DR4, death receptor 4; DR5, death receptor 5; CD95, Fas receptor/cluster of differentiation 95; FADD, Fas-associated protein with death domain; TRADD, tumor necrosis factor receptor type 1-associated death domain; DISC, death-inducing signaling complex; BID, BH3 interacting-domain death agonist; tBID, truncated BH3 interacting domain death agonist; Bcl-2, B-cell lymphoma 2; Bcl-xL, B-cell lymphoma extra-large; Mcl-1, myeloid cell leukemia 1; BIM, Bcl-2-interacting mediator of cell death; BAD, Bcl-2 associated agonist of cell death; BMF, Bcl-2-modifying factor; Noxa, gene PMAIP1 (phorbol-12-myristate-13-acetate-induced protein 1); PUMA, p53 upregulated modulator of apoptosis; MOMP, mitochondrial outer membrane permeabilization; BAX, Bcl-2-associated X protein; BAK, Bcl-2 homologous antagonist/killer; Apaf-1, apoptotic protease activating factor 1; SMAC, second mitochondria-derived activator of caspases; OMI, high temperature requirement factor A2 (HtrA2); XIAP, X-linked inhibitor of apoptosis protein; WD40, WD40 repeats; CARD, caspase recruitment domain; Ca^2+^, calcium ions.

Additionally, a presence of negative regulation of caspase function is critical due to the proteolytic irreversible process and possible inappropriate cell destruction. This process can be achieved by IAPs including X-linked IAP (XIAP), survivin, which exerts anti-apoptotic activity against activation of executor caspases-9, -3, and -7 [70]. On the contrary, a cytosolic release of SMAC or OMI mitochondrial proteins can suppress the effect of IAP through the binding to baculovirus IAP repeat (BIR) domain causing the reduction of caspases activity [71]. The extrinsic and intrinsic apoptotic pathways are illustrated in Figure 2.

### 3.3. Other Mechanisms Involved in Regulation of Apoptosis

There is a growing awareness of the role of epigenetic mechanisms in the regulation of cell death. Epigenetic modifications including miRNA expression [72,73,74], methylation of DNA [75], or chemical modifications of histones are responsible for the control of gene expression [10,76,77,78] that can trigger apoptosis via intrinsic or extrinsic pathways.

Moreover, a deregulation of apoptosis can be associated with changes in metabolic profiles and current evidence suggests that metabolic switch plays a role in the survival of tumor cells or metastatic development during carcinogenesis [9]. Since its very first description almost 100 years ago, the Warburg-effect has been connected to glucose metabolism and therefore represent one of the most accepted postulates in terms of cancer physiopathology [79].

In addition, lipid metabolism with specific lipid molecules such as peroxisome proliferator-activated receptor γ (PPARγ) and/or ceramides are intrinsically related to apoptotic mechanisms [80,81], gene expression regulating cellular differentiation [82], and carcinogenesis [83,84].

Furthermore, reactive oxygen species (ROS) can be involved in mitochondrial, death receptor and endoplasmic reticulum pathways of apoptosis. Low doses of ROS regulate different physiological functions involved in the development, including cell cycle progression, proliferation, differentiation, migration and cell death [85,86]. On the other hand, high doses of ROS induce various damages of proteins, nucleic acids, lipids, membranes and organelles resulting in the activation of apoptotic signaling [86,87]. As a result of damaged cells by ROS, various specific signaling pathways are involved in the apoptosis, including Ras/mitogen-activated protein kinases (MAPKs) pathway [88,89], extracellular signal-regulated kinases (ERKs), jun kinases (JNKs) and p38 MAPK [90] p38 and JNK MAPK pathways [91]. MAPK pathway steps (ASK1, MEKK1, MEKK2, MEKK3, MEKK4, and MLK3) [92], TNF superfamily members is mediated by ROS [93].

Ras-dependent pathway (activation of nuclear factor-kappa-light-chain-enhancer of activated B cells (NF-κB)) [94]. Janus kinase/signal transducer and activators of transcription (JAK/STAT) signaling [95,96,97], phosphoinositide 3-kinase/protein kinase B (PI3K)/Akt) signaling pathway [98,99], p53 [100], cyclin-dependent kinase inhibitor (p21) [101], and inhibitor of cyclin-dependent kinases (p27 kip1 (p27)) [102,103].

## 4. Carotenoids

**Figure 3:**
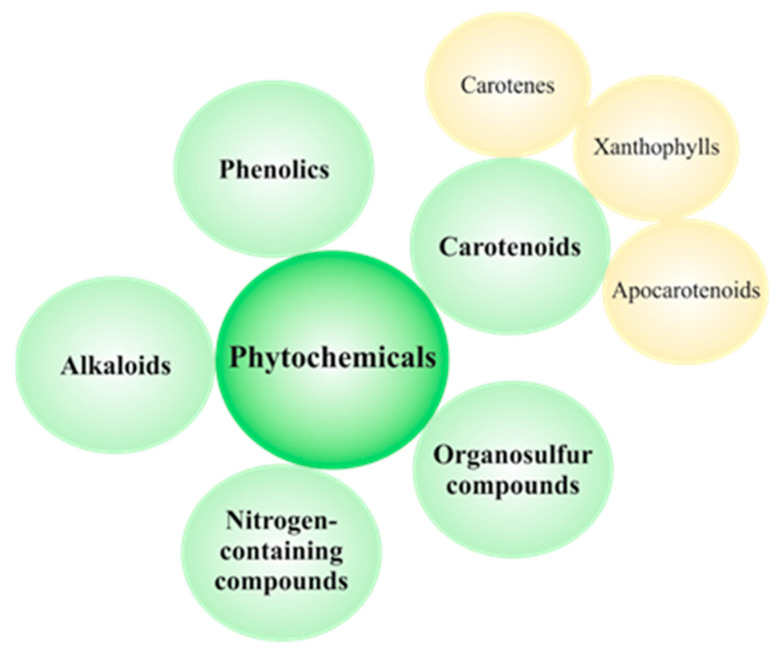
Classification of dietary phytochemicals. Phytochemicals are classified into five groups including phenolics, alkaloids, nitrogen-containing compounds, organosulfur compounds, and carotenoids. Carotenoids are divided into carotenes, xanthophylls, and apocarotenoids.

Vegetables, fruits, and whole grains are rich in natural compounds called phytochemicals. Due to the dietary origin, phytochemicals are presumed to be safer and better tolerated thanks to relatively low toxicity [104]. Therefore, plant-based foods rich in different bioactive phytochemicals can reduce the risk of various diseases including cancer [105,106]. Thousands of individual phytochemicals have been identified and can be classified as carotenoids, phenolics, alkaloids, nitrogen-containing compounds, and organosulfur compounds [106]. The classification of dietary phytochemicals is summarized in Figure 3.

**Figure 4:**
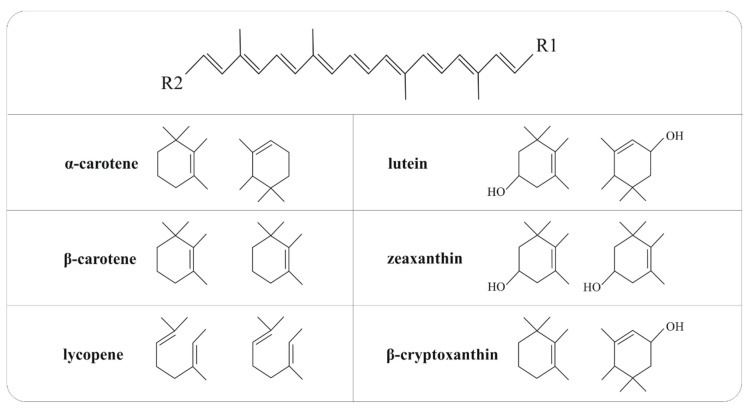
The chemical structure of major representatives of carotenoids. The main part of the carotenoid structure includes a central carbon chain with alternating single and double bonds and various cyclic or acyclic end groups (R1 and R2), which vary in individual carotenoids.

Carotenoids belong to the tetraterpenes family with a parent C_40_H_56_ hydrocarbon skeleton conjugated by alternating single and double bonds with photochemical properties and chemical reactivity. Moreover, the central carbon chain carries different cyclic or acyclic end groups. Besides, very low solubility of carotenoids in water is related to their structure as the lipophilic and hydrophobic compounds [14,107,108]. Chemical structures of the main carotenoid representatives are illustrated in Figure 4.

Carotenoids can be classified into two groups: carotenes and xanthophylls, which are well known due to their characteristic colors in a range of yellow to red spectrum [109,110] and exert beneficiary effects on human health, including the prevention of human diseases or maintenance of good health by known anti-inflammatory and antioxidant effects and enhancement of the immune system [16,17,109,111]. Various plants, algae, fungi, birds, fish flesh, and cuticle of crustaceans or insects are considered as a source of these natural pigments [14]. Aside from dietary carotenoids, xantophylls such as astaxanthin, is usually found in various bacteria, microalgae, and yeasts [15], fucoxanthin is presented in marine algae and diatom [112,113], and fucoxanthinol, deacetylated metabolite of fucoxanthin, is found in bacteria or marine diatom [114,115]. Apocarotenoids are compounds such as retinoids, vitamin A, β-ionone, α-ionone, and aromatic volatile compounds derived from carotenoids by oxidative cleavage via dioxygenases [110]. Vitamin A is an essential micronutrient that is present in multiple forms including retinols, retinals or retinoic acids with special functions in the regulation of growth and differentiation of many cell types [116].

Humans and animals are not able to synthesize carotenoids that are essential for their physiological processes; therefore, carotenoids have to be obtained from the adapted diet intake [117]. So far, more than 700 carotenoids have been identified in Nature [13]; however, only about 40 carotenoids are usually present in the human diet [111]. Carotenes such as α-carotene, β-carotene, and lycopene and xanthophylls, including lutein, zeaxanthin, and β-cryptoxanthin are the main representatives of carotenoids commonly found in the human diet, as described in Table 1.

## 5. The Use of Carotenoids in Preclinical Cancer Research

Recently, not only dietary carotenoids but also carotenoids from other sources have attracted attention in cancer research, especially for their ability to inhibit tumor growth and induce apoptosis [107,120]. Moreover, carotenoids are useful not only for cancer treatment but also for cancer prevention [23,24].

### 5.1. Carotenoids-Induced Apoptosis in In Vitro and In Vivo Studies

Apoptosis, one of the hallmarks of cancer, is a promising target in anticancer therapy. Plant-derived foods rich in phytochemicals can alter the intracellular and extracellular signals with subsequent activation of apoptosis in cancer cells [8]. The same effect is proven in carotenoids derived from sources other than food [114,121]. As will be described below, several studies have revealed that carotenoids can induce apoptosis through the modulation of various apoptotic molecules and pathways.

#### 5.1.1. Carotenes

##### β-Carotene

β-carotene isolated from *Spinacia oleracea* induced apoptosis in MCF-7 human breast adenocarcinoma cells. β-Carotene increased the activity of intracellular caspase-3 associated with condensed nuclei and nuclear fragmentation in comparison to control cells. Moreover, β-carotene decreased the expression of Bcl-2, NF-κB, and poly(ADP-ribose) polymerase (PARP) [122], an enzyme involved in the cellular response to DNA damage and detections of DNA strand breaks [123]; however, changes in the expression of the pro-apoptotic proteins such as BAX or p53 were not detected. Besides, β-carotene inhibited ERK1/2 and Akt while decreased Akt correlated with decreased phosphorylation of BAD protein at Ser136 in the same in vitro model [122]. Similarly, β-carotene at lower concentrations induced apoptosis in TE1 human esophageal squamous cell carcinoma cells (ESCS) and higher concentrations in EC1 and Eca109 ESCS cells compared to Het-1A healthy esophageal epithelial cells. The BAX/Bcl-2 ratio also remarkably increased after β-carotene treatment. Moreover, β-carotene increased the expression of caspase-3 and decreased the p-Akt and p-NF-κB protein levels suggesting that the apoptosis can be regulated through the Akt/NF-κB pathways [124]. Interestingly, a combination of β-carotene and a chemotherapeutic agent 5-fluorouracil (5-FU) synergistically induced apoptosis in both in vitro and in vivo models of ESCS. Treatment with β-carotene and 5-FU decreased Bcl-2 level and increased BAX and caspase-3 level in Eca109 ESCS cells and also Eca109 mice xenografts when compared with either agent alone. Additionally, the protein level of Caveolin-1 (Cav-1), p-Akt, p-NF-κB, phosphorylated mammalian target of rapamycin (p-mTOR), and phosphorylated ribosomal protein S6 kinase beta-1 (p-p70S6K) was significantly reduced after the combination of β-carotene and 5-FU compared to 5-FU alone [125]. Moreover, β-carotene, doxorubicin/luteolin and doxorubicin-treated cells revealed upregulated apoptosis due to pronounced pro-oxidant action in MCF-7 and MDA-MB-231 breast cancer cells while non-significant cytotoxicity was observed in normal breast epithelial cells exposed to similar treatment [126]. In addition, the percentage of apoptotic cells increased with increased β-carotene concentration, especially in Huh7 hepatoma cells [127]. Also, β-carotene induced increase in ROS and caspase-3 activity that may reduce the level of DNA repair Ku proteins resulting in the apoptosis of AGS gastric cancer cells [128]. On the contrary, β-carotene pre-treatment exerted antioxidant effects against oxidative stress in K562 human erythromyeloblastoid leukemia cells protecting them against the damage by oxidative stress [129].

##### Lycopene

Lycopene, a red-colored non-oxygen-containing carotenoid, inhibits cell proliferation and subsequently enhances apoptosis in a dose-dependent manner. In SKOV3 ovarian cancer cells this was demonstrated through the upregulation of BAX and downregulation of Bcl-2 [130]. Noteworthily, mitochondrial dysfunction, which is determined by the mitochondrial membrane potential, is the reason for the generation of ROS leading to the rapid growth of pancreatic cancer cells [31,131]. However, apoptosis caused by lycopene was associated with a higher level of caspase-3 and increased BAX/Bcl-2 ratio in PANC-1 pancreatic cancer cells. Treatment with lycopene triggered apoptosis in PANC-1 pancreatic cancer cells through a decreased level of intracellular and mitochondrial ROS, suppressed NF-κB activation and its targeting gene expressions of cellular inhibitors of apoptosis (cIAP1 and cIAP2), and survivin [31]. Moreover, lycopene induced apoptosis through increased DNA fragmentation, caspase-3, and caspase-9 cleavage, and BAX/Bcl-2 ratio increase in AGS gastric cancer cells. Apoptosis was also associated with an inhibition of the intracellular and mitochondrial ROS levels and suppression of activation of the ROS-mediated EGFR/Ras/ERK and p38 MAPK pathways. Subsequently, these processes led to the decreased DNA-binding activity of NF-κB p50/p50 homodimer and gene expression of cyclooxygenase-2 (COX-2) [132]. Additionally, a lower level of pyruvate kinase isozyme M2 (PKM2) promotes cell proliferation through the entering carbohydrate metabolites of glycolysis into alternative pathways, which produce macromolecules or nicotinamide adenine dinucleotide phosphate (NADPH) necessary for tumor growth [133]. Lycopene treatment increased the level of proteins associated with the induction of apoptosis such as PKM2, tyrosyl-tRNA synthetase (TyrRS), and 40S ribosomal protein S3 (RPS3) in PrE prostatic epithelial cells. Moreover, lycopene downregulated anti-apoptotic proteins including intracellular chloride channel protein 1 (CLIC1), heat shock protein 70 1A/1B (HSP70 1A/1B), HSP27, Rho GDP-dissociation inhibitor 1 (Rho GDI 1), translationally controlled tumor protein (TCTP), lactoylglutathione lyase, 78 kDa glucose-regulated protein (Grp78), and protein kinase C inhibitor protein 1 (KCIP1) in PrE cells. In the view of signaling pathways, downregulation of thioredoxin domain containing protein 17 (TXNDC17) after lycopene treatment in PrE cells enhanced TNF-*a* that activated caspase and then apoptosis. Similarly, stimulation of Akt/mTOR pathway by epithelial cell marker protein 1 (SFN) decreased after lycopene treatment. Besides, lycopene upregulated tumor suppressor N-Myc downstream regulated 1 (NDGR1) activating p53/TP53-mediated caspase and apoptosis in PrE cells [134]. Furthermore, lycopene induced apoptosis of oral cancer cells by deactivating PI3K/Akt/m-TOR signaling via an increase in BAX and downregulation of p-PI3K, p-Akt, p-m-TOR, and Bcl-2 [135]. Also, lycopene promoted apoptosis of MCF-7 breast cancer cells in vitro probably through upregulation of p53 and BAX expression [136]. In addition, lycopene extracts from different tomato-based food products induced apoptosis of human primary prostate cancer cells, upregulated TP53 and BAX, and downregulated Bcl-2 [137].

#### 5.1.2. Xanthophylls

##### Lutein

Lutein induced minimal late apoptosis and necrosis in MDA-MB-468 breast cancer cells but not in MCF-7 breast cancer cells, however, the early apoptosis was not altered. Besides, apoptosis in MDA-MB-468 and MCF-7 cells was mediated through the caspase-independent mitochondrial pathway. Treatment with lutein in MDA-MB-468 cells revealed increased expression of pro-apoptotic genes (growth arrest and DNA damage inducible alpha (GADD45A), BAX, caspases-3/4/8, TNF receptor superfamily member 10a (TNFRSF10A), and TNF receptor superfamily member 21 (TNFRSF21)) except for cluster of differentiation 70 (CD70) that decreased together with anti-apoptotic gene Bcl-2. Additionally, higher phosphorylation of p53 associated with increased HSP60 levels was detected in MDA-MB-468 cells, which explain the growth inhibitory effects of lutein [32]. Furthermore, deregulation of PI3K/Akt signaling pathway is common in cancerous conditions. Lutein triggered apoptosis by an inhibition of PI3K/Akt signaling pathway in A549 lung cancer cells with no side effects [138]. In addition, lutein induced apoptosis of MDA-MB-157 and MCF-7 breast cancer cells [139]. Similarly, lutein promoted apoptosis in MDA-MB-231 and MCF-7 breast cancer cells with increased caspase-3 activity and downregulated Bcl-2 and poly-ADP ribose polymerase [140].

##### β-Cryptoxanthin

β-Cryptoxanthin, one of the six major carotenoids, induced apoptosis in in vitro and in vivo models of gastric cancer. An increased expression of cleaved caspase-3, -8, and -9 and cytochrome c was observed in β-cryptoxanthin treated AGS and SGC-7901 gastric cancer cells. Moreover, the decreased levels of protein kinase A (PKA), phosphorylated AMP-activated protein kinase (pAMPK), and eukaryotic elongation factor 2 kinase (eEF2k) after β-cryptoxanthin treatment led to the inactivation of AMPK signaling in the same gastric cancer cells. The induction of apoptosis by β-cryptoxanthin was accompanied by G0/G1 cell cycle arrest. Similarly, the induction of apoptosis by β-cryptoxanthin at the doses of 5 and 10 mg/kg was observed in AGS mice xenografts resulting in a suppression of tumor growth [25]. Furthermore, β-cryptoxanthin in cooperation with oxaliplatin, a third-generation platinum-based chemotherapeutic drug, was found to induce apoptosis via negative regulation of a dominant-negative inhibitor of wild-type p53 and Tap73 (ΔNP73) in HCT116, SW480-ADH, and SW1417 colon cancer cells [141].

##### Astaxanthin

An experimental study revealed that the treatment with astaxanthin increased BAX and caspase-3 expression and decreased Bcl-2 expression, leading to the induction of apoptosis and inhibition of growth and proliferation of LS-180 colorectal cancer cells. The efficacy of astaxanthin on LS-180 cell apoptosis correlated with the antioxidant activity of astaxanthin associated with the reduction of the malondialdehyde levels and increased levels of antioxidant enzymes, including superoxide dismutase, catalase, and glutathione peroxidase [142]. Furthermore, an intragastrical administration of astaxanthin in PC-3 prostate cancer cells mice xenografts demonstrated antitumor potential. A higher dose of astaxanthin (100 mg/kg) triggered apoptosis associated with an increased level of apoptotic cancer cells as well as with elevated expression of caspase-3; however, the differences between a lower dose of astaxanthin (25 mg/kg) and control group were not detected [143]. The effects of astaxanthin on cell cycle arrest closely related to the apoptosis were also demonstrated in the study of human colon cancer. Different stereoisomers of astaxanthin such as S (from *Haematococcus pluvialis*), R (from *Phaffia rhodozyma*), and mixture of S:meso:R (1:2:1) (isolated from synthetic astaxanthin) induced apoptosis of HCT-116 and HT-29 colon cancer cells in time- (24–72 h) and dose- (4–16 μM) dependent manner; however, without significant changes between tested stereoisomers. Additionally, cell cycle arrest at G2/M phase induced by different stereoisomers of astaxanthin was mediated via increased levels of p21Cip1/Waf1, p27, and p53, and lower levels of cyclin-dependent kinases (CDK4 and CDK6). Also, the induction of apoptosis was linked with the activation of caspase-3 and PARP in HCT-116 cells. These comparative study of different stereoisomers of astaxanthin revealed that various terminal ring structures might not be the main factor affecting the antitumor activity of astaxanthin [144]. In addition, astaxanthin from shrimp with β-carotene and lutein from greens synergistically induced apoptosis through modulation of cyclin D1, p53, BAX, and Bcl-2 expression in MCF-7 breast cancer cells [145]. Besides, astaxanthin induced cell cycle arrest of mice H22 hepatoma cells in vitro and in vivo and had only little impact on apoptosis [146].

##### Fucoxanthin

The chemopreventive mouse model of lung cancer induced by benzo(a)pyrene (B(a)P) demonstrated the significant role of fucoxanthin in the prevention of cancer development. Antitumor effects were accompanied by decreased progression of B(a)P-induced lung cancer in mice, increased antioxidant activity and apoptosis, decreased tumor markers, and regulation of apoptotic molecules. The apoptosis was detected by increased expression of caspase-9 and -3 and decreased expression of Bcl-2 in mice treated with fucoxanthin compared to controls [147]. In another study, fucoxanthin purified from the marine microalgae *Nitzschia* sp. inhibited tumor growth by an induction of apoptosis in U251 human glioma cells. Fucoxanthin induced ROS-mediated DNA damage as an early apoptotic event and consequently increased the levels of PARP and caspase-3 in dose- and time- dependent manner in U251 cells. Besides, fucoxanthin caused the dysfunction of MAPKs and PI3K-Akt pathways that resulted in increased phosphorylation of Thr183-JNK, Thr180-p38, and Thr202-ERK and decreased phosphorylation of Ser473-Akt [121]. TRAIL demonstrated an ability to trigger apoptosis selectively in tumors but not in healthy cells [148]. A combination of fucoxanthin and TRAIL showed a strong synergistic effect targeting the apoptosis in SiHA human cervical cancer cells more than fucoxanthin or TRAIL alone. Similarly, increased expression of BAX and decreased expression of Bcl-2 was more significant after the combination fucoxanthin plus TRAIL compared to single substances in SiHa cells. Interestingly, fucoxanthin significantly inhibited the PI3K/Akt/NF-κB pathway activation induced by TRAIL in SiHa cells. The results pointed out the importance of a combination drug therapy approach within cancer research [149]. Moreover, fucoxanthin inhibited viability, induced autophagy and apoptosis through upregulation of beclin-1, microtubule-associated proteins 1A/1B light chain 3 (LC3), and cleaved caspase-3, and downregulation of Bcl-2 in SGC7901 gastric cancer cells [150]. Furthermore, fucoxanthin activated apoptosis via inhibition of PI3K/Akt/mTOR pathway in U87 and U251 human glioma cancer cells [151] and induced apoptosis in adenocarcinoma SGC-7901 or BGC-823 human gastric cells involving downregulation of Mcl-1, STAT3 and p-STAT3 [152].

##### Fucoxanthinol

The antitumor activities of brown algae, a traditional Japanese food with a high content of carotenoids, especially fucoxanthin (FX) and its deacetylated metabolite known as fucoxanthinol (FXOH), have also attracted attention in cancer research [153,154]. FX and FXOH showed cytotoxic effects against HCT-116 human colon cancer cells in dose- and time-dependent manner. A comparative study between FX and FXOH demonstrated the more potent apoptotic activity of FXOH at lower concentrations (10 μM) when compared with FX at higher concentrations (25 μM). The treatment with FXOH (48 h) rapidly increased the SubG1 cell population (a marker of running apoptosis) up to 62% vs. control. Moreover, compared to FX, FXOH showed higher apoptotic activity via its ability to inhibit NF-κB transcriptional activity and suppress X-linked IAP family genes [154]. Additionally, yellow pigment fraction isolated from *Pseudomonas stutzeri* JGI 52 with the high content of carotenoids, mostly fucoxanthinol, revealed antitumor potential in HeLa cervical cancer cells and HepG2 hepatocellular carcinoma cells. This carotenoid fraction triggered DNA fragmentation in both tested cell lines. About a 6-7-fold increased activity of caspase-3 in HeLa and HepG2 cells compared to untreated control cells was detected after 48 h exposure to yellow pigment fraction. The authors suggested that the fucoxanthinol as the primary representative in yellow pigment fraction from *P. stutzeri* JGI 52 represents the responsible inductor of apoptosis in tested cancer cells [114].

##### Deinoxanthin

A carotenoid isolated from the radioresistant bacterium *Deinococcus radiodurans* called deinoxanthin triggered apoptosis in time-dependent manner via chromatin condensation and nuclear fragmentation in HepG2 hepatoma, HT-29 colon, and PC-3 prostate cancer cells. Moreover, in all tested cell lines, the authors observed decreased Bcl-2 expression and increased BAX and caspase-3 expression after deinoxanthin treatment. Additionally, higher ROS production was detected in HepG2, HT-29, and PC-3 cells suggesting that ROS could be a mediator of deinoxanthin-induced apoptosis [155].

#### 5.1.3. Apocarotenoids

##### β-Ionone

The treatment with β-ionone derived from β-carotene, multi-kinase inhibitor sorafenib, or their combination exerted pro-apoptotic effects mediated through increased BAX and reduced Bcl-2 expression in diethylnitrosamine (DENA)-induced hepatocellular carcinoma of Wistar rats. Moreover, all treated groups were associated with upregulated gene expression of PPAR-γ and forkhead box protein O1 (FOXO-1) [156] that are involved in the regulation of various cellular processes, including apoptosis [157,158]. Above all, a combination of sorafenib and β-ionone demonstrated more significant pro-apoptotic activity in studied in vivo model of hepatocellular carcinoma compared to sorafenib or β-ionone alone [156]. Furthermore, β-ionone induced apoptosis in MCF-7 breast cancer cells through increased expression of cleaved caspase-3 and cytochrome c in a dose-dependent manner. Indeed, decreased Bcl-2 and higher BAX expression in MCF-7 cells were observed only after a higher dose of β-ionone. However, the BAX/Bcl-2 ratio increased time-dependently after β-ionone treatment of MCF-7 cells. Besides, β-ionone promoted apoptosis-associated expression of p-P38 from the MAPK-P38 pathway in a dose-dependent manner [159].

##### Crocetin

Crocetin, an apocarotenoid derived from the traditional Chinese medical herb saffron dose-dependently triggered mitochondrial-mediated apoptosis in KYSE-150 esophageal cancer cells through the disruption of mitochondrial membrane potential, increase in BAX and caspase-3 and decrease in Bcl-2 levels. Additionally, inhibition of PI3K/Akt activation, ERK1/2, p38, and upregulation of p53/p21 levels indicated the suppression of cell growth and induction of apoptosis after crocetin treatment of KYSE-150 cells [160]. Moreover, crocetin induced apoptosis of HCT-116 colon cancer cells independently of p53 status. Further evaluation revealed that crocetin targets p53-mediated activation of BAX and death domain protein (PIDD)/caspase-2-mediated activation of BID that together converge to mitochondria and cause the loss of transmembrane permeability and activation of caspase cascade in p53-expressing HCT-116 cancer cells. On the contrary, functional p53-impaired HCT-116 cells can utilize alternative p73-FAS-FADD-caspase-8 axis leading to mitochondrial disruption and apoptosis. Taken together, due to the significant pro-apoptotic efficacy of crocetin in p53-expressing as well as p53-impaired cancer cells, apoptosis targeted by plant-derived compounds should represent a novel strategy in the therapy of malignant diseases, especially human colon cancer [161].

##### Crocin

Crocin, a less water-soluble apocarotenoid also derived from saffron, demonstrated pro-apoptotic activity in A431 and SCL-1 human skin cancer cells. Crocin caused G0/G1 cell cycle arrest leading to apoptosis through increased expression of BID and procaspase-3 and decreased expression of Bcl-2 in both cell lines. Besides, the mechanism of apoptosis could be related to the downregulation of the JAK/STAT signaling pathway [162]. Crocin also triggered the apoptosis of Y79 and WERI-RB-1 retinoblastoma cells through cleaved PARP and induced cleavage of pro-caspase-3 into its active form. Interestingly, the pro-apoptotic efficacy of crocin in Y79 cells was dependent on MYCN, a member of the MYC family, which encodes the N-myc proto-oncogene protein. Despite that the treatment with crocin reduced the expression and stability of MYCN in Y79 cells, MYCN overexpression reverted the effects of crocin [163]. Impressively, crocin demonstrated the pro-apoptotic activity in chemosensitive OV2008 and chemoresistant C13 cervical cancer cells through an increased level of BAX and p53 and a decreased level of Bcl-2 and miR-365 that is an upstream regulator of both BAX and Bcl-2; however, slightly better pro-apoptotic effect of crocin was observed in OV2008 sensitive cells. Results suggested the role of crocin as an adjuvant agent to elude the resistance and improve the treatment of cervical cancer [34]. Moreover, crocin in combination with hyperthermia revealed pro-apoptotic efficacy in MDA-MB-468 breast adenocarcinoma cells via an increase of BAX/Bcl-2 ratio and decreased expression of apoptosis-related HSP70 and HSP90 [164]. Similarly, apoptotic modulation of MCF7 breast cancer cells was attributed to *Crocus sativus* extract (CSE) and its major constituent crocin through time-dependent decrease of Bcl-2, upregulation of BAX, dowregulation of caspase-8 and -9 and cleaved caspase-3 [165]. Additionally, simultaneous use of crocin and radiation could increase the sensitivity of radiation as was demonstrated by in vitro study in which crocin decreased cell viability, induced sub-G1 peak, and sensitized HN-5 head and neck cancer cells to radiation-induced toxicity and apoptosis [166].

##### Picrocrocin

Picrocrocin, like crocin, is derived from saffron through the biosynthetic pathway [167]. Picrocrocin treatment promoted ROS production and decreased the mitochondrial membrane potential of SK-MEL-2 melanoma cells that led to the induction of apoptosis and cell cycle arrest. Furthermore, inhibition of JAK/STAT5 pathway was also demonstrated in picrocrocin-treated SK-MEL-2 cells [168].

##### Bixin

Bixin, a Food and Drug Administration (FDA)-approved natural food colorant [169] and the main apocarotenoid present in *Bixa orellana* seeds promoted apoptosis of A2058 human melanoma cells via higher capsase-3 cleavage. Moreover, bixin in combination with dacarbazine, an alkylating agent used in melanoma therapy, increased the activity of caspase-3 in A2058 cells suggesting their potential synergistic effects in cancer treatment. Besides, bixin sensitized A2058 cells to dacarbazine through increased ROS production and DNA damage that allowed the entrance of the dacarbazine into A2058 cells and promoted the apoptosis [170]. In the study of human hepatocarcinoma, bixin induced apoptosis in Hep3B cells through a higher level of ROS and loss of mitochondrial membrane potential compared to control. Moreover, apoptosis of bixin-treated Hep3B cells was related to the increased protein level of BAX, FasL and cleavage of caspase-9, -8 and -3 that suggests an involvement of both extrinsic and intrinsic apoptotic pathways. In addition, a docking study revealed energetically favorable binding potential between bixin and BAX or Fas proteins [171].

##### Retinoids

All-trans retinoic acid (ATRA) is a derivative of vitamin A that enhances the cytotoxicity of multitargeted kinase inhibitor sorafenib in HepG2 hepatocellular carcinoma cells. Importantly, ATRA-induced AMPK activation leading to reduced intracellular adenosine triphosphate (ATP) level and downregulation of mRNA level of glucose transporter (GLUT-1), PKM2, and lactate dehydrogenase A (LDHA) contributed to the mitochondrial apoptotic pathway in the tested cell line. Moreover, apoptosis induced by ATRA + sorafenib was associated with activation of p38 MAPK and JNK, increased BAX and caspase-3 in HepG2 cells [33]. Furthermore, fenretinide, a synthetic retinoid, demonstrated pro-apoptotic activity through increased level of ROS and ceramide in HL60 acute promyelocytic leukemia cancer cells. However, the inhibition of apoptosis occurred after the combinatorial treatment with fenretinide and ceramide synthase inhibitor fumonisin B1. Additionally, fumonisin B1 reverted the loss of mitochondrial transmembrane potential after fenretinide-induced apoptosis in HL60 cells suggesting that apoptotic activity of fenretinide was dependent on ceramide [172]. In summary, above-mentioned extensive preclinical studies, summarized in Table 2, provide the insight into the effects of carotenoids in anticancer research due to their abilities to inhibit tumor growth and induce cell cycle arrest, mainly in terms of apoptotic induction. Besides, the understanding of the role of carotenoids in molecular mechanisms and apoptotic signaling pathways can give the base for their further testing and application in clinical practice, especially in high-risk individuals.

### 5.2. Nanotechnology and Carotenoids

Existing deficits in anticancer therapy include insufficient specificity, biodegradation and rapid drug clearance, and inadequate targeting [176]. To this end, nanotechnology-based therapeutical targeting is instrumental to compensate those deficits. According to the International Union of Pure and Applied Chemistry (IUPAC), nanoparticles (NP) are defined as small particles at the size level of 100 nm or less [177]. Due to microscopic size, large surface to volume ratio and modifiable surface chemistry, NPs manifest beneficial properties such as longer circulation time, easy penetration into the cell, and effective site-specific targeting [178]. On the other hand, complications such as the toxicity of NPs are barriers that have to be overcome when considering their utility in the practical nanomedicine. Recently, a combination of natural products such as carotenoids with NPs demonstrates a promising way to reduce toxicity in healthy cells and accelerate apoptosis in cancer [179].

In the following section of the presented review, we summarized studies that evaluated the effects of nanotechnology associated with carotenoids in the modulation of the apoptotic cascades in experimental cancer research. Jain et al., analyzed β-carotene loaded zein nanoparticles to improve biopharmaceutical properties and to eliminate the toxicity of chemotherapeutic agent methotrexate. The acquired data revealed that co-delivery of β-carotene NPs with methotrexate had the highest apoptotic index followed by β-carotene + methotrexate, plain methotrexate, β-carotene NPs, and free β-carotene. Hence, the results of selective apoptosis suggested synergic effect β-carotene NPs with methotrexate in MCF-7 cells [180].

Similarly, Langroodi et al., analyzed co-delivery of poly (lactic-co-glycolic acid) (PLGA) NPs loaded with crocetin (natural carotenoid) and anthracycline antibiotic doxorubicin. Obtained data verified the potency of co-delivered doxorubicin and crocetin loaded PLGA NP that showed a more potent anticancer effect on MCF-7 cells. Furthermore, PLGA encapsulated doxorubicin and crocetin upregulated caspase-3 expression in MCF-7 cells [181]. Interestingly, crocetin-loaded PLGA NPs significantly increased the induction of apoptosis compared to crocetin without NPs formulation in MCF-7 cells. Therefore, the application of NPs loaded crocetin can eliminate the disadvantage of the carotenoid such as poor solubility that causes limitations in further clinical application [182]. Nanogels are defined as nanoparticles composed of cross-linked hydrophilic polymer networks with a capability to form homogenous colloidal solutions [183]. Fucoxanthin is a marine carotenoid isolated from macroalgae and microalgae. Due to its lipophilicity, fucoxanthin exerts poor bioavailability. Chitosan (CS) combined with glycolipid (GL) nanogels (NGs) enhanced the anticancer activity of fucoxanthin in Caco-2 colon cancer cells. Fucoxanthin loaded in NGs + GL with CS induced apoptosis through the suppression of Bcl-2, upregulation of BAX levels, and promotion of caspase-3 activity in analyzed cancer cells [184]. 

Additionally, nanostructured lipidic carriers (NLCs) coated with CS and loaded with fucoxanthin manifested potential to regulate apoptosis via the downregulation of Bcl-2 in psoriatic cells. Although the study investigated non-cancerous cells, the acquired results showed the great potential of NLCs + CS + fucoxanthin, which could be applied also in cancer research [185]. Moreover, lycopene nanogold nanoemulsion significantly reduced the expression of procaspases-3, -8, and -9, Bcl-2 and PARP-1 in HT-29 colon cancer cells. Additionally, the expression of BAX was significantly elevated compared to the control group or groups treated only with lycopene or gold nanoparticles separately [186]. Similarly, lycopene loaded solid lipid nanoparticles (lycopene-SLNs) combined with methotrexate (MTX) revealed anticancer efficacy mediated via the induction of apoptosis in MCF-7 cancer cells. In this regard, the synergic effect of the well-established anticancer drug and nanoparticle loaded lycopene is associated with excellent opportunities in anticancer therapy [187]. In another study, astaxanthin-alpha tocopherol nanoemulsion manifested anticancer features via the reduction of cell viability and induction of apoptosis in HeLa, CT26, and T24 cells [188]. The antitumor potential of astaxanthin nanoemulsion was also shown via the induction of mitochondrial-mediated apoptosis in vitro. The application of nanoemulsion resulted in apoptosis and ROS generation in AGS and HT-29 cells [189]. Furthermore, hydroxylated tetraterpenoid deinoxanthin isolated from *D. radiodurans* was used for the synthesis of gold nanoparticles (AuNPs) and to evaluate its efficacy against cancer MCF-7 cells. The acquired data showed that deinoxanthin-AuNPs induced ROS generation, DNA damage, and apoptosis in cancer cells. Analysis of gene expressions revealed the upregulation of 374 genes and downregulation of 135 genes associated with cellular processes, including autophagy and apoptosis induced by deinoxanthin-AuNPs [190]. Last but not least, histone deacetylase inhibitor trichostatin A (TSA) and graphene oxide-silver nanoparticles (rGO-AgNPs) synthesized using lycopene caused ROS generation, DNA damage, mitochondrial dysfunction, and thus induced apoptosis in SKOV3 ovarian cancer cells [191]. All the above mentioned studies are summarized in Table 3. In summary, nanotechnology in a combination with carotenoids brings new possibilities in the treatment of various cancer types. Obtained data from above-described studies demonstrate an enormous potential of nanomedicine in cancer management. However, clinical studies focused on carotenoids in combination with nanotechnology are still absent. Only more in-depth research in the clinical sphere can bring new hope for individuals suffering from cancer.

## 6. Carotenoids in Clinical Research

The effects of carotenoids in the prevention or treatment of cancer is intensively investigated in clinical research. Despite the evaluation of the role of carotenoids in cancer prevention in the second half of 20th century [192,193,194] their positive and negative effects in cancer chemoprevention and therapy have been supported by a great amount of more recent evidence.

Several studies demonstrated anti-cancer efficacy of carotenoids. Diet rich in lycopene and β-cryptoxanthin may protect against aggressive prostate cancer among European-American and African-American men included in the North Carolina-Louisiana prostate cancer project, respectively [195]. Similarly, Hung et al., observed protective effects of plasma carotenoids on bladder cancer [196]. Moreover, increased α-tocopherol (and not β-carotene or retinol) led to increased overall survival of prostate cancer survivals [197]. Interestingly, a combination of carotenoids and myo-inositol was related to the prevention of hepatocellular carcinoma in patients with chronic viral hepatitis and cirrhosis [198]. In addition, an inverse association between certain plasma carotenoids and tocopherols and prostate-specific antigen (PSA) levels suggested potentially beneficial effects of these micronutrients to men with PSA-defined prostate cancer recurrence [199]. Due to the contribution of increased oxidative stress, diet low in antioxidants can indirectly contribute to the breast cancer recurrence. Therefore, Thomson et al., evaluated the role of plasma and dietary carotenoids in the reduction of oxidative stress in women previously treated for breast cancer. Significant inverse association was observed between total plasma carotenoid concentrations and oxidative stress measured by urinary 8-hydroxy-2’-deoxyguanosine (8-OHdG) while only modest significance was shown for 8-iso-prostaglandin F2α (8-iso-PGF2α). Nevertheless, the protective abilities were not related to dietary carotenoid intake [200].

However, results of other studies have shown no significant protective effects of carotenoids in cancer prevention or management. Long term supplemental α-tocopherol or β-carotene revealed no effect on liver cancer or chronic liver disease mortality [201]. No association was observed between dietary antioxidant vitamins and carotenoids and risk of colorectal cancer in male smokers [202]. In addition, Dorgan et al., demonstrated no relation between the risk of development of basal cell carcinoma and serum levels of any measured carotenoid or α-tocopherol [203]. Importantly, two week intervention with carotenoid-rich carrot and tomato juice in twenty-two healthy young men was associated only with minor changes in luminal biomarkers relevant to colon carcinogenesis [204].

In addition to no significant protective effect, other authors demonstrated rather negative impact of carotenoids on the process of carcinogenesis. In 1996, Omenn et al., demonstrated that the combination of β-carotene and vitamin A revealed no benefit and may have had adverse effects on the incidence of lung cancer and risk of death from lung cancer, cardiovascular disease as well as any cause in smokers and workers exposed to asbestos [205]. More recently, β-carotene supplementation was associated with increased risk of lung cancer in smokers while this risk is not dependent upon the tar or nicotine level of smoked cigarettes suggesting the recommendation of all smokers to avoid β-carotene supplementations [206]. Similarly, higher serum retinol concentrations were related to greater risk of prostate cancer in male smokers over a 20-year period [207].

### Carotenoids in Clinical Research Targeting Apoptosis

Regarding the effects of carotenoids on the processes associated with apoptosis of cancer cells in human studies, the available evidence is already modest or deficient. An evaluation of differential effects of lycopene consumed in tomato paste and lycopene in the form of a purified extract on target genes of cancer prostatic cells was conducted on thirty healthy men assigned to two groups. Eventually, cells incubated with sera from men who consumed red tomato paste in comparison with sera collected after the first washout period revealed a significant upregulation of BAX/Bcl-2 ratio and downregulation of p53 that are both implicated in the apoptosis as well as upregulation of insulin-like growth factor-binding protein (IGFBP), which is also associated with the regulation of apoptosis through the Bcl-2 protein family [208]. Moreover, a topical treatment of basal cell carcinoma (BCC) with new acetylenic retinoid selective for retinoic acid receptor (RAR)-b and c isotypes, tazarotene, revealed more than 50% clinical and dermoscopic regression in 70.8% of BCC and 30.5% healed BCC without recurrences after 3 years of follow-up. The biopsy showed that the regression was associated with reduced proliferation and increased apoptosis of basaliomatous cells [209]. Additionally, results of the study conducted on twenty-six men with newly diagnosed prostate cancer demonstrated that lycopene might decrease the growth of prostate cancer; however, no significant changes were observed in the expression of markers of apoptosis, Bcl-2, and BAX [210].

Above all, the effects of carotenoids in the process of carcinogenesis in general or their effects on apoptotic pathways in clinical trials need to be further analyzed because of diverse outcomes across different types of cancer or populations.

Carotenoids have demonstrated important roles in the processes of apoptotic induction, scavenging free radicals, or inhibition of angiogenesis in various cancer types [211]. Despite protective effects of carotenoids demonstrated in preclinical and clinical studies, their applicability in clinical practice is associated with limitations. Detrimental interactions appear to exist between carotenoids, especially β-carotene and specific populations such as cigarette smokers who were associated with increased risk of lung or prostate cancer [205,206,207,212]. However, the use of carotenoids is complicated by the lack of their solubility in water due to their glycosylated form [211]. Therefore, poor absorption and bioavailability is a disadvantage associated with the introduction of carotenoid-rich food into the diet [213].

Above all, the effects of carotenoids in the process of carcinogenesis in general or their effects on apoptotic pathways in clinical trials need to be further analyzed because of diverse outcomes across different types of cancer or populations. Thus, well-defined clinical studies are needed to obtain more accurate results defining effects and mechanisms of action of carotenoids in the prevention of malignant disease or their role in cancer treatment. At the same time, the ever-advancing progress in research brings further insights into increasing the content and bioavailability of carotenoids.

## 7. The Content and Bioavailability of Carotenoids

Carotenoids included in the human diet are associated with health beneficiary efficacy. Regarding the content of carotenoids in food sources, 100% watermelon juice was demonstrated to effectively increase serum lycopene in older women belonging to the group of people that are at risk for low carotenoid intake [214]. However, the disadvantage of the carotenoids is their poor absorption and bioavailability [213]. An illustrative example of the bioavailability of carotenoids can be demonstrated by an example of phytoene, phytofluene, lycopene, and β-carotene that represent the main tomato carotenes. An evaluation of carotene bioavailability in mice after gavage with carotene-rich oil-in-water emulsion revealed significantly higher bioavailability of phytofluene in comparison with other carotenes. The determination of its absorption profile showed the proximal and distal intestine as the site of conversion of β-carotene while phytoene and phytofluene accumulation tended to be more important in the distal intestine [215].

### 7.1. The Effects of Cooking Method on the Content of Carotenoids

As has been demonstrated by recent studies, the content of carotenoids in the food is influenced by several factors. Three cooking methods (boiling kernels, porridge, and tortilla) were evaluated in order to determine the content change of carotenoids in maize. After all, tortilla and porridge were found to be better dietary choices for an intake of xanthophylls and β-carotene among maize-based foods while the content of xanthophylls influenced the absorption of β-carotene regardless the cooking method [216]. However, the content of carotenoids in meals was not related to nutrition-optimizing cooking practice, as quantified by the Healthy Cooking Index (HCI) scores or total fruit and vegetable usage while high-carotenoid meals appeared to be a result of the use of canned tomato products. Moreover, high-carotenoid content dishes were also high in sugar and refined grains. Nevertheless, increased carotenoid intake for childhood cancer survivors could mitigate the late-effects of cancer treatment such as cardiovascular disease or secondary cancers. However, further research is needed in order to elucidate the role of carotenoids in this population [217]. Besides, cooking with olive oil was associated with health-improving effects of the Mediterranean diet due to the detected polyphenols in the olive oil after the cooking as well as the high content of carotenoid *Z*-isomers that are more available than *E*-isomers. Moreover, oil added to tomato products could partly help to dissolve lycopene that is otherwise in insoluble crystalline form and protect it from thermal oxidation during the cooking process. Fat also improves the absorption of carotenoids [218]. On the contrary, the cooking process decreased total lutein, which was observed to be the main carotenoid compound in raw samples of pigmented rice [219].

### 7.2. Current Trends in the Bioavailability of Carotenoids

The research aimed to increase the bioavailability of carotenoids is currently yielding significant results. Based on recently published studies performed in experimental in vitro and in vivo conditions as well as in clinical sphere, a wide range of possibilities influencing the content and bioavailability of carotenoids in the organism exists.

### 7.3. The Effects of Dietary Constituents on Carotenoid Bioavailability

Dietary constituents or co-consumption of other food can significantly affect the bioavailability of carotenoids. Citrus flavanones, hesperetin and hesperidin, improved β-carotene incorporation efficiency, intestinal cell uptake, and retinoid concentrations in tissues. On the contrary, the flavanones naringenin and naringin reduced the incorporation efficiency of β-carotene and decreased hepatic retinoid concentrations after 7 days of gavage [220]. Importantly, lycopene in tomatoes is mostly present in the all-E-configuration that is associated with very low bioavailability, while Z-isomers are related to higher bioavailability. However, some food ingredients including vegetable (*Allium* sp., *Brassica* sp., and *Raphanus* sp.), shiitake mushroom (*Lentinus edodes*), and edible seaweeds (*Saccharina* sp. and *Ecklonia* sp.) markedly promoted *Z*-isomerization of (all-*E*)-lycopene in tomato puree with heating at 80 °C for 1 h [221].

Similarly, higher bioavailability and/or liver accumulation of Z-isomers of lycopene than *E*-isomer was demonstrated in mice [222]. Furthermore, exopolysaccharides from milk fermented by lactic acid bacteria enhanced dietary carotenoid bioavailability in humans and rats. The dietary β-carotene absorption could be enhanced via simple diffusion mechanisms mediated by exopolysaccharides affecting the physical properties of fermented milk [213]. The improved bioavailability of carotenoids, due to the other food constituents or co-consumption is shown Table 4.

### 7.4. Biofortification as a Way to Increase the Content and Bioavailability of Carotenoids

Biofortification is attracting increased interest as a method to increase the carotenoid content of staple foods [223]. A randomized, crossover, placebo-controlled trial revealed that β-cryptoxanthin and zeaxanthin are highly bioavailable from whole-grain and refined biofortified orange maize in humans with optimal status of vitamin A [224]. An association between lettuce genotypes and thermal treatments on β-carotene and lutein bioaccesibility to the micellar fraction was evaluated using human Caco-2 cells model. Thermal treatment of lettuce leaves increased the bioavailability of carotenoids and absorption of β-carotene and lutein. However, the food matrix disruption by prior cooking reduced carotenoid levels and transfer to the micellar fraction. Therefore, absorption of carotenoids from biofortified lettuce is similar to low carotenoid lettuce cultivars due to the disrupted food matrix [225]. The role of biofortification in the increase of the content and bioavailability of carotenoids is shown in Table 5. 

### 7.5. Solid Dispersion and Microemulsions as a Mean of Increased Solubility and Bioavailability of Carotenoids

The low solubility of β-carotene in rats was improved via an amorphous state induced by solid dispersion prepared by hot-melt technology with polyvinylpyrrolidone and sucrose fatty acid esters. The importance of this technology is associated with improvements in the solubility of β-carotene without organic solvents with potential use in pharmaceutical as well as food industry [226]. Similarly, β-carotene solid dispersion prepared by hot-melt technology, which were found to have an amorphous structure, improves its solubility in water [227]. Moreover, possible increase in β-carotene and lycopene bioaccessibility was associated with directly processing microemulsions from pitanga (*Eugenia uniflora*) and buriti (*Mauritia flexuosa*) fruits that are very rich in carotenoids [228]. The detailed overview of solid dispersion and microemulsions as processes of increased bioavailability of carotenoids is shows in Table 6.

Additionally, an amount of other extrinsic (molecular structure and environmental factors such as interactions with drugs, smoking or alcohol intake) and intrinsic (age, body composition, hormonal fluctuations or variations in genes) factors affect the physiologic response to carotenoids, which highlights the gaps in the current knowledge and the need for further research [229].

## 8. The Utility Potential of Carotenoids in the Framework of 3P Medicine

As detailed above, carotenoids are natural agents demonstrating multi-level bioactivities applicable for complementary medicinal purposes against cancer development and progression in general. In particular, nanomedical constructs based on bioactive substances derived from carotenoids are currently under extensive consideration for targeted therapeutic approaches. Contextually, detailed patient stratification is essential to increase the efficacy of corresponding therapeutic approaches. Most prominent examples are listed below.

### 8.1. The Level of General Cancer Prevention

Plant natural modulators have been demonstrated as being effective in general cancer prevention [230]. Dietary habits with well-balanced carotenoids intake adapted to individual profiles are strongly recommended distinguishing between individual age groups in population, sedentary life-style versus regular physical exercise and high level of sport activities, increased versus mild hormonal and metal stress load, amongst others [231,232,233]. The level of individually shifted (balanced against imbalanced) stress reactions can be monitored utilizing well established technology of comet assay analysis applied at sub-cellular level [4,234].

### 8.2. The Level of Cancer Prevention in Stratified Groups at Increased Cancer Risk

Several groups in the population have been reported as being at increased risk for cancer development such as shift workers, obese persons, individuals with “dry mouth” syndrome, vulva-vaginal dryness, and/or Sicca syndrome, low grade inflammation, systemic hypoxic conditions and ischemic lesions e.g., characteristic for vasospastic individuals (e.g., Flammer Syndrome phenotype), amongst others [235,236,237]. Although featuring cancer risks in common, they demonstrate group-specific particularities and some obviously dominating triggers of risks (e.g., imbalanced stress load, systemic dehydration and/or dryness, chronic inflammation, shifted expression profiles of relevant molecular pathways etc.) to be carefully considered for adapted treatment algorithms aimed at the cost-effective targeted prevention [238]. Application of carotenoids-based nanoceria as a prebiotic for stratified patients can be considered [239].

### 8.3. Cancer Prevention in Relevant Syndromes and Comorbid Conditions

Imbalanced stress reactions characteristic for metabolic stress in patients diagnosed with diabetes mellitus (DM) type 1 and type 2 are well-known risk factors for development of several cancer types such as pancreatic malignancies, endometrium and liver carcinoma, amongst others [240]. Frequently linked to DM but also as an independent comorbid condition, impaired healing is a strong contributor to the cancer development [241]. Patients diagnosed with the DM and/or impaired healing, have been demonstrated as strongly benefiting from adapted dietary supplements including carotenoids [242,243,244].

Sjögren Syndrome related to Sicca syndrome is another prominent group of well stratified patients with clear cancer risks, where dietary supplements including carotenoids may play a crucial role in the disease prevention focused on immune system impairments and chronic inflammation as specific targets [245].

### 8.4. Secondary Chemoprevention of Metastatic Disease

Cytostatic properties of carotenoids are of great importance for the chemopreventive approach against metastatic disease. For example, the incidence of both metastatic breast and prostate cancers is on the rise over the last decades, which makes an effective chemoprevention particularly important for healthcare providers [234,246]. To this end, one of the evident deficits is the reactive character of medical services currently provided. Consequently, predictive and prognostic liquid biopsy tests for circulating tumor cells (CTC) enumeration and specific molecular patterns in blood are considered to be useful for secondary prevention of metastatic disease [234,247,248].

At any level of the targeted cancer prevention, individualized patient profiling and multi-level diagnostics are instrumental for disease prediction, patient stratification and personalization of medical services [238,249,250,251].

## 9. Conclusions and Expert Recommendations

Based on data obtained from in vitro and in vivo studies, carotenoids seem to be a good choice for cancer therapy and prevention that have apparent potential to improve the efficacy of conventional treatment or reduce the risk in highly predisposed individuals. The experimental and epidemiological data strongly indicate that carotenoids represent perspective candidates for the anti-cancer therapeutics, however, their effective and safe using in clinical practice deserve additional in-depth evaluations. In this regard, several important issues must be resolved: (1) carotenoid levels evaluated in preclinical experimental approaches are much higher than those in human blood, therefore, improving the bioavailability of carotenoids in organism using progressive methods such as nanotechnology are needed, (2) data about other pharmacokinetic parameters (e.g., absorption, excretion) of numerous carotenoids are necessary, (3) identification of the effective and safe doses of specific carotenoids for clinical application with respect to long-term cancer treatment and prevention because epidemiological studies indicated certain undesirable effects of higher doses in humans, (4) analyses of the drug combination approaches over single drug applications with the aim to decrease the dosages of the conventional medications, (5) evaluation of synergistic action of low concentrations of various carotenoids that is usually more effective than each carotenoid alone, (6) analyzing if described impacts of carotenoids on various signaling pathways in the cell are caused by direct effects of the carotenoids or are mediated by their derivatives/metabolites, (7) determination of the effective and biologically active carotenoid metabolites, (8) overcoming the acquired resistance against treatment by carotenoids that can re-sensitize the chemotherapy/radiotherapy-resistant cancers, (9) analyzing the anti-cancer potential of carotenoids regarding the reduction of cancer cell invasion, metastasis, and chances of disease relapse, (10) later, target mechanisms associated with the individual characteristics could be better understand with the aim to develop more personalized medications based on carotenoids. Targeted application of carotenoids to stratified patients groups demonstrated a great potential to contribute to the concepts of 3P medicine [252]. 

The “from bench to bedside” concept describes the rapid transfer of findings from laboratory research into practical application on patients. Moreover, the concept “from bench to bedside and back” uses the experiences and knowledge gained by doctors with their patients for laboratory research that can improve the diagnostics and therapeutics [253,254]. The anti-cancer effects of β-carotene might be used as a good example used in mentioned concept. Preclinical studies revealed positive impact of β-carotene in vitro and in vivo [125,127,128]. Similarly, positive effects of β-carotene were observed in clinical practice but only in non-smoker individuals. In specific population such as cigarette smokers the use of β-carotene increases the risk of lung or prostate cancer [205,206,207,212]. These findings lead to the return of β-carotene testing to the laboratory with the aim to find the differences between cigarette smoke-exposure animals compared to cigarette nonsmoke-exposure animals. Unfortunately, clinical results are often irrelevant and might be determined again in preclinical research. Precise determination of anti-cancer effects of carotenoids and other natural substances in preclinical and clinical practice, their use in diagnostics and treatment in wide group of individuals can give us the better knowledge of their effects.

## Figures and Tables

**Table 1:** Major representatives of carotenoids in food.

Carotenoids	Food Source	Reference
α-Carotene	carrots, coleslaw, pumpkin	[19,111]
β-Carotene	apricot, beans, beet, blueberry, broccoli, brussels sprouts, carrots, celery, coleslaw, courgetti, cucumber, lettuce, mango, parsley, peas, pepper, plum, pumpkin, spinach, watermelon, melon, grape, spring greens, watercress	[19,111,118]
Lycopene	tomatoes, watermelon	[19,111]
Lutein/zeaxanthin	beans, beet, broccoli, brussels sprouts, carrots, celery, coleslaw, courgetti, cucumber, kiwi, leeks, lettuce, parsley, peas, pepper, pumpkin, sweetcorn, olive oil, spinach, watercress, egg, kale, asparagus, pistachio nuts	[19,111,118,119]
β-Cryptoxanthin	oranges, pepper, tangerine, papaya	[14,19]

**Table 2:** The effects of carotenoids on cancer apoptosis.

Carotenoids Group	Carotenoids	Study Design	Effects on Apoptosis	Reference
Carotenes	β-Carotene	MCF-7 human breast cancer cells	↑ caspase-3, ↓ Bcl-2, ↓ PARP, ↓ NF-κB, ↓ BAD at Ser136, ↓ Akt and ERK1/2 activation	[122]
		TE1, EC1, Eca109 human esophageal squamous cell carcinoma cells	↑ caspase-3, ↓ Bcl-2, ↓ p-NF-κB, ↓ p-Akt, ↓ Akt/NF-κB pathways	[124]
		Huh7 hepatoma cells	↑ percentage of apoptotic cells with an increasement of β-carotene concentration	[127]
		AGS gastric cancer cells	↑ ROS, ↑ caspase-3, ↓ DNA repair Ku proteins	[128]
	β-Carotene pretreatment	K562 human erythromyeloblastoid leukemia cells	↑ antioxidant effects against oxidative stress, ↑ protection against the damage of oxidative stress	[129]
	β-Carotene, doxorubicin/luteolin, doxorubicin-treated cells	MCF-7 and MDA-MB-231 breast cancer cells	↑ apoptosis, ↑ prooxidant action	[126]
	β-Carotene and 5-fluorouracil	Eca109 human esophageal squamous cell carcinoma cells and Eca109 mice xenografts	↓ Bcl-2, ↑ caspase-3, ↑ BAX, ↓ Cav-1, ↓ p-Akt, ↓ p-NF-κB, ↓ p-mTOR, ↓ p-p70S6K	[125]
	Lycopene	SKOV3 ovarian cancer cells	↓ Bcl-2, ↑ BAX	[130]
		PANC-1 pancreatic cancer cells	↑ caspase-3, ↑ BAX/Bcl-2 ratio, ↓ intracellular and mitochondrial ROS, ↓ NF-κB, ↓ cIAP1, ↓ cIAP2, ↓ survivin	[31]
		AGS gastric cancer cells	↑ DNA fragmentation, ↑ caspase-3, -9 cleavage, ↑ BAX/Bcl-2 ratio, ↓ EGFR/Ras/ERK, ↓ p38 MAPK, ↓ DNA-binding activity of NF-κB p50/p50 homodimer ↓ COX-2 expression	[132]
		PrE prostatic epithelial cells	↑ PKM2, ↑ TyrRS, ↑ RPS3, ↓ CLIC1, ↓ HSP70 1A/1B, ↓ HSP27, ↓ Rho GDI 1, ↓ TCTP, ↓ lactoylglutathione lyase, ↓ Grp78, ↓ KCIP1, ↓ TXNDC17, ↑ TNF-*a*—induced caspases and apoptosis, ↓ Akt/mTOR, ↓ SFN, ↑ NDGR1, ↑ p53/TP53-mediated caspase and apoptosis	[134]
		Oral cancer cells	↓ PI3K/Akt/m-TOR signaling, ↑ BAX, ↓ p-PI3K, ↓ p-Akt, ↓ p-m-TOR, ↓ Bcl-2	[135]
		MCF-7 breast cancer cells	↑ p53, ↑ BAX	[136]
	Lycopene extracts from different tomato-based products	Human primary prostate cancer cells	↑ TP53, ↑ BAX, ↓ Bcl-2	[137]
Xanthophylls	Lutein	MDA-MB-468 and MCF-7 breast cancer cells	↑ GADD45A, ↑ BAX, ↑ caspase-3, -4, -8, ↑ TNFRSF10A, ↑ TNFRSF21, ↓ CD70, ↓ Bcl-2, ↑ p53 phosphorylation, ↑ HSP60	[32]
		A549 lung cancer cells	Regulation of PI3K/Akt signaling pathway	[138]
		MDA-MB-157 and MCF-7 breast cancer cells	↑ apoptosis	[139]
		MDA-MB-231 and MCF-7 breast cancer cells	↑ caspase-3, ↓ Bcl-2, ↓ poly-ADP ribose polymerase	[140]
	β-Cryptoxanthin	AGS and SGC-7901 gastric cancer cells; AGS mice xenografts	↑ caspase-3, -8, and -9, ↑ cytochrome c, ↓ PKA, ↓ pAMPK, ↓ eEF2k, ↑ AMPK signaling inactivation, ↑ G0/G1 cell cycle arrest	[25]
	β-Cryptoxanthin + oxaliplatin	HCT116, SW480-ADH, and SW1417 colon cancer cells	↑ apoptosis via negative regulation of ΔNP73	[141]
	Astaxanthin	LS-180 colorectal cancer cells	↑ BAX, ↑ caspase-3, ↓ Bcl-2, ↑ antioxidant activity, ↓ malondialdehyde, ↑ superoxide dismutase, ↑ catalase, ↑ glutathione peroxidase	[142]
		PC-3 prostate cancer cells mice xenografts	↑ caspase-3	[143]
		mice H22 hepatoma cells	↑ cell cycle arrest, little impact on apoptosis	[146]
	Astaxanthin + β-carotene + lutein	MCF-7 breast cancer cells	↑ apoptosis through modulation of cyclin D1, p53, BAX, and Bcl-2	[145]
	Stereoisomers of astaxanthin (*S, R*, *S:meso:R* 1:2:1)	HCT-116 and HT-29 colon cancer cells	↑ caspase-3, ↑ PARP, ↑ G2/M cell cycle arrest, ↑ p21Cip1/Waf1, ↑ p27, ↑ p53, ↓ CDK4, ↓ CDK6	[144]
	Fucoxanthin	Chemopreventive mouse model of B(a)P-induced lung cancer	↑ antioxidant activity, ↑ caspase-9 and -3, ↓ Bcl-2	[147]
		U251 human glioma cells	↑ ROS-mediated DNA damage, ↑ PARP, ↑ caspase-3, ↑ dysfunction of MAPKs and PI3K-Akt pathways, ↑ phosphorylation of Thr183-JNK, Thr180-p38, and Thr202-ERK, ↓ phosphorylation of Ser473-Akt	[121]
		SGC7901 gastric cancer cells	↓ viability, ↑ autophagy, ↑ apoptosis, ↑ beclin-1, ↑ LC3, ↑ cleaved caspase-3, ↓ Bcl-2	[150]
		U87 and U251 human glioma cancer cells	↓ PI3K/Akt/mTOR pathway	[151]
		SGC-7901 or BGC-823 human gastric cells	↓ Mcl-1, ↓ STAT3, ↓ p-STAT3	[152]
	Fucoxanthin + TRAIL	SiHa human cervical cancer cells	↑ BAX, ↓ Bcl-2, ↓ PI3K/Akt/NF-κB pathway	[149]
	Fucoxanthinol	HCT-116 colon cancer cells	↑ SubG1 population, ↑ NF-κB transcriptional activity	[173]
	Yellow pigment fraction (fucoxanthinol)	HeLa cervical cancer cells and HepG2 hepatocellular carcinoma cells	↑ DNA fragmentation, ↑ caspase-3	[114]
	Deinoxanthin	HepG2 hepatoma, HT-29 colon, and PC-3 prostate cancer cells	↑ chromatin condensation, ↑ nuclear fragmentation, ↓ Bcl-2, ↑ BAX, ↑ caspase-3, ↑ ROS production	[155]
Apocarotenoids	β-Ionone + sorafenib	Diethylnitrosamine-induced hepatocellular carcinoma of Wistar rats	↑ BAX, ↓ Bcl-2, ↑ PPAR-γ, ↑ FOXO-1	[156]
	β-Ionone	MCF-7 breast cancer cells	↑ caspase-3, ↑ cytochrome c, ↓ Bcl-2, ↑ BAX, ↑ BAX/Bcl-2 ratio, ↑ p-P38	[159]
	Crocetin	KYSE-150 esophageal cancer cells	↓ mitochondrial membrane potential, ↑ BAX, ↑ caspase-3, ↓ Bcl-2, ↓ PI3K/Akt activation, ↓ ERK1/2, ↓ p38, ↑ p53/p21	[160]
		p53-expressing and p53-impaired HCT-116 colon cancer cells	p53-expressin HCT-116 cells: ↑ BAX, ↑ PIDD, ↑ BID cleavage, ↑ caspase-2, ↓ mitochondrial membrane potential, ↑ caspase-9 and -3 p53-impaired HCT-116 cells: switch-over from p53 to p73, ↑ FAS, ↑ BID, ↑ FAS-FADD-caspase-8-pathway.	[161]
	Crocin	A431 and SCL-1 human skin cancer cells	↑ G0/G1 cell cycle arrest, ↑ BID, ↑ procaspase-3, ↓ Bcl-2, ↓ JAK/STAT signaling pathway	[162]
		Y79 and WERI-RB-1 retinoblastoma cells	↑ PARP, ↑ caspase-3, ↓ MYCN but the overexpression of MYCN revert the inhibitory effect of crocin	[163]
		Sensitive OV2008 and chemoresistant C13 cervical cancer cells	↑ BAX, ↑ p53, ↓ Bcl-2, ↓ miR-365	[34]
		MCF7 breast cancer cells	↓ Bcl-2, ↑ BAX, ↓ caspase-8 and -9, ↓ cleaved caspase-3	[165]
	Crocin + hyperthermia	MDA-MB-468 breast adenocarcinoma cells	↑ BAX/Bcl-2 ratio, ↓ apoptosis-related HSP70 and HSP90	[164]
	Crocin + radiation	HN-5 head and neck cancer cells	↑ sensitivity of radiation, ↓ cell viability, ↑ sub-G1 peak, and sensitized cancer cells to radiation-induced toxicity and apoptosis	[166]
	Picrocrocin	SK-MEL-2 melanoma cells	↑ ROS production, ↓ mitochondrial membrane potential, ↓ JAK/STAT5 pathway	[168]
	Bixin + dacarbazine	A2058 human melanoma cells	↑ caspase-3, ↑ ROS production, ↑ DNA damage	[170]
	Bixin	Hep3B human hepatocarcinoma cells	↑ ROS level, ↓ mitochondrial membrane potential, ↑ DNA damage, ↑ BAX, ↑ FasL, ↑ caspase-9, -8 and -3, binding interaction between BAX and Fas ligand	[171]
	All-trans retinoic acid	HepG2 hepatocellular carcinoma cells	↑ AMPK, ↓ intracellular ATP level, ↑ p38 MAPK, ↑ JNK, ↑ BAX, ↑ caspase-3, ↓ GLUT-1, *↓* PKM2, *↓* LDHA	[33]
	Fenretinide	HL60 acute promyelocytic leukemia cancer cells	Fenretinide-induced apoptosis: ↑ ROS, ↑ ceramide Fenretinide with fumonisin B1: ↓ apoptosis, reversion of the loss of mitochondrial transmembrane potential	[172]
The whole plant rich in canrotenoids	Lipophilic extracts of tomatoes (San Marzano and Corbarino)	YCC-1, YCC-2 and YCC-3 gastric cancer cells	↑ G0/G1 cell cycle arrest, ↑ RBL2/p130, ↑ PARP expression, ↑ caspase-9, ↑ p27, ↑ p21	[174]
	Zucchini (“Yellow” and “Light Green”)	HL60 human promyelocytic leukemia cells	↑ DNA fragmentation and apoptosis	[175]

Explanatory notes: ↑, increase/induce; ↓, decrease/inhibit. Abbreviation: Bcl-2, B-cell lymphoma 2; PARP, poly (ADP-ribose) polymerase; NF-κB, nuclear factor-κB; BAD, Bcl-2 associated agonist of cell death; Akt, protein kinase B; ERK, extracellular signal-regulated kinases; Cav-1, caveolin-1; mTOR, mammalian target of rapamycin; p-p70S6K, ribosomal protein S6 kinase beta-1/p70S6 kinase; cIAP1, cellular inhibitor of apoptosis protein-1; cIAP2, cellular inhibitor of apoptosis protein-2; EGFR, epidermal growth factor receptor; Ras; member of small GTPase; MAPK, mitogen-activated protein kinase; COX-2, cyclooxygenase-2; PKM2, pyruvate kinase isozymes M2; TyrRS, tyrosyl-tRNA synthetase; RPS3, 40S ribosomal protein S3; CLIC1, chloride intracellular channel protein 1; HSP70 1A/1B, heat shock 70 kDa protein 1A/1B; HSP27, heat shock protein 27; Rho GDI 1, Rho GDP-dissociation inhibitor 1; TCTP, translationally controlled tumor protein; Grp78, 78 kDa glucose-regulated protein; KCIP1, protein kinase C inhibitor protein 1; TXNDC17, thioredoxin domain containing protein 17; TNF-*a*, tumor necrosis factor-alpha; SFN, stratifin/epithelial cell marker protein 1; NDGR1, N-Myc Downstream Regulated 1; GADD45A, growth arrest and DNA damage inducible alpha; TNFRSF10A, TNF receptor superfamily member 10a; TNFRSF21, TNF receptor superfamily member 21; CD70, cluster of differentiation 70; HSP60, heat shock protein 60; PI3K, phosphoinositide 3-kinases; PKA, protein kinase A; AMPK, AMP-activated protein kinase; eEF2k, eukaryotic elongation factor-2 kinase; p21Cip1/Waf1, cyclin-dependent kinase inhibitor 1 or CDK-interacting protein 1; CDK4, cyclin-dependent kinase 4; CDK6, cyclin-dependent kinase 6; ROS, reactive oxygen species; JNK, c-Jun N-terminal kinase; PPAR-γ, peroxisome proliferator-activated receptor gamma; FOXO-1, forkhead box protein O1; PIDD, P53-induced death domain; BID, BH3 interacting-domain death agonist; FAS, Fas cell surface death receptor; FADD, Fas-associated protein with death domain; MYCN, MYCN proto-oncogene, BHLH transcription factor; JAK/STAT, Janus kinase/signal transducers and activators of transcription; FasL, Fas ligand; ATP, adenosine triphosphate; GLUT-1, glucose transporter 1; LDHA, lactate dehydrogenase A; RBL2/p130, retinoblastoma-like/p130.

**Table 3:** An overview of studies focused on carotenoids combined with nanotechnologies and their impact on the modulation of apoptosis.

Carotenoid	Carriers System	Experimental Model	Effect	Reference
β-Carotene	Zein nanoparticles	MCF-7 breast cancer cells	↑ apoptotic activity	[180]
Crocetin	PLGA	MCF-7 breast cancer cells	↑ caspase-3	[181]
	PLGA	MCF-7 breast cancer cells	↑ apoptosis	[182]
Fucoxanthin	CS + NGs + GL	Caco-2 colorectal cancer cells	↓ Bcl-2; ↑ BAX; ↑ caspase-3 activity	[184]
	CS + NLCs	Psoriatic-like cellular model	↓ Bcl-2	[185]
Lycopene	Nanoemulsion carrying gold nanoparticles	HT-29 colorectal cancer cells	↓ procaspase -3, -8, -9; ↓ Bcl-2; ↓ PARP-1	[186]
	Solid lipid nanoparticles	MCF-7 breast cancer cells	↑ greater rate of apoptosis in combination with MTX	[187]
	rGO-AgNPs	SKOV3 ovarian cancer ells	↑ apoptosis	[191]
Astaxanthin	Nanoemulsion	HeLa cervical cancer cells, CT26 colon cancer cells, T24 transitional cell carcinoma cells	↑ apoptosis, ↓ cell viability	[188]
	Nanoemulsion	HT-29 colorectal cancer cells, AGS gastric cancer cells	↑ apoptosis, ↑ ROS generation	[189]
Deinoxanthin	AuNPs	MCF-7 breast cancer cells	Regulation of genes associated with apoptosis and autophagy	[190]

Explanatory notes: ↑, increase/induce; ↓, decrease/inhibit. Abbreviations: PLGA, poly (lactic-co-glycolic acid); CS, chitosan; NGs, nanogels; GL, glycolipid; NLCs, nanostructured lipidic carriers; AuNPs, gold nanoparticles; rGO-AgNPs, graphene oxide-silver nanoparticles; MTX, methotrexate.

**Table 4:** The role of food constituents in the bioavailability of carotenoids.

Purpose of the Study	Study Details	Results	Reference
The effects of flavanones co-consumption (hesperetin, hesperidin, naringenin, and naringin) on β-carotene	Experimental models: in vitro digestion procedure, synthetic mixed micelles, Caco-2 cell monolayers, and gavage experiments in mice	Hesperetin (25 μM) and hesperidin (25 μM) standards: significant increase in the incorporation efficiency of the β-carotene standard to 68.7 ± 3.6 and 75.2 ± 7.5% (*p* < 0.05) Naringenin (25 μM) and naringin (25 μM) standards: Significant reduce in the incorporation efficiency of β-carotene by 23.8 and 26.4% (*p* < 0.05).	[220]
		β-carotene cellular absorption in the Caco-2 cell model (scavenger receptor class B type I expression increase promoted by citrus flavanones)	
		Citrus flavanones (7.5 mg kg-1 day-1) increased the retinoid concentrations in tissues (after 3 days of gavage) Naringenin and naringin significantly decreased hepatic retinoid concentrations (*p* < 0.05) (after 7 days of gavage)	
Enhanced Z-isomerization of tomato lycopene through food ingredients (*Allium* sp., *Brassica* sp., and *Raphanus* sp., *Lentinus edodes*, *Saccharina* sp. and *Ecklonia* sp.)		Promotion of Z-isomerization of (all-E)-lycopene in tomato puree with heating at 80 °C for 1 h	[221]
		Enhanced thermal Z-isomerization of (all-E)-lycopene by polysulfides, isothiocyanates, carbon disulfide, iodine (commonly contained in the above food ingredients)	
Bioavailability and/or liver accumulation of lycopene (Z-isomer)	Mice	Higher bioavailability and/or liver accumulation vs. E-isomer	[222]
Bioavailability of β-carotene (through exopolysaccharides from milk fermented by lactic acid bacteria)	Male rats (*n* = 8/group) administered with β-carotene or β-carotene + fermented milk. Male rats (*n* = 6/group) retreated with ezetimibe (investigation of β-carotene transport mechanism)	Serum β-carotene AUC significantly higher for the β-carotene + fermented milk vs. β-carotene only	[213]
		Significant correlation between the exopolysaccharide content of fermented milk and serum β-carotene AUC was observed	
		Ezetimibe treatment did not suppress elevations in serum β-carotene concentrations induced by fermented milk ingestion	
	Three studies using a randomized crossover method (*n* = 16/study) consumed a vegetable (carrot, tomato, or spinach) drink alone or with a fermented milk drink (UMIN000034838, UMIN000034839, UMIN000034840)	Significantly higher iAUC for β-carotene in plasma TRL fraction (carrot + fermented milk vs. carrot drink alone)	
		Significantly higher iAUC for lycopene in the plasma TRL fraction (tomato + fermented milk vs. tomato drink alone)	
		Significant increase in plasma lutein in all fractions after consumption of spinach + fermented milk and not with spinach drink alone	

Abbreviations: AUC, area under the concentration-time curve; iAUC, incremental area under the concentration-time curve; TRL, triacylglycerol-rich lipoprotein.

**Table 5:** The impact of biofortification on the content and bioavailability of carotenoids.

Purpose of the Study	Study Details	Results	Reference
Bioavailability of BCX and zeaxanthin from whole-grain and refined BCX-biofortified maize vs. white maize	Randomized, crossover, placebo-controlled trial: 9 adults (mean ± SD age: 23.4 ± 2.3 y; 5 men) were provided with muffins made from BCX-enhanced WGOM, ROM or RWM	Significantly higher BCX AUC for WGOM and ROM vs. RWM	[224]
		Greater increase in serum BCX from WGOM muffins (131%) than from ROM muffins (108%)	
		Higher Zeaxanthin AUCs for WGOM and ROM vs. RWM	
Biofortified lettuce varieties (effects of thermal treatment on carotenoids)	Caco-2 cells	Thermal treatment of lettuce leaves increased carotenoid availability (higher lutein and β-carotene absorption)	[225]
		Thermal disruption of the food matrix by prior cooking reduced carotenoid levels and transfer to the micellar fraction (absorption of carotenoids from biofortified lettuce remained similar to lettuce cultivars with low carotenoid levels)	

Abbreviations: AUC, area under the concentration-time curve; BCX, β-Cryptoxanthin; ROM, refined orange maize; RWM, refined white maize; WGOM, whole-grain orange maize.

**Table 6:** The impact of solid dispersion and microemulsions on the solubility and bioavailability of carotenoids.

Purpose of the Study	Study Details		Results	Reference
Solubility of β-carotene	Solid dispersion prepared by hot-melt technology with polyvinylpyrrolidone and sucrose fatty acid esters	Rats	High solubility	[226]
	Hot-melt technology (solid dispersions technology)—the weight ratio of β-carotene:polyvinylpyrrolidone:sucrose fatty acid ester to 10%:70%:20%		Improved water solubility of β-carotene	[227]
Bioaccessibility of β-carotene, lycopene	Pitanga (*E. uniflora*) and buriti (*M. flexuosa*) microemulsions: direct processing (high-speed homogenization at 15,000 rpm and ultrasound with 20 kHz probe at 40% amplitude) of the whole pulp together with surfactant (Tween 80 or Whey Protein Isolate at 2%) and corn oil (5%)	Dynamic gastrointestinal system (simulation of human digestion)	Surfactant and oil: protection of carotenoids in fruits and microemulsions	[228]
			Final recovery of total carotenoids, higher for microemulsions than for whole pulps	
			High losses of total carotenoids in buriti and β-carotene and lycopene in pitanga during jejunum and ileum phases	
